# Anti-pruritic effect of L-carnitine against chloroquine-induced pruritus mediated via nitric oxide pathway

**DOI:** 10.1186/s40360-024-00748-4

**Published:** 2024-05-22

**Authors:** Kiran Seemab, Arif-ullah Khan, Muhammad Imran Khan, Neelum Gul Qazi, Amber Mahmood Minhas, Fawad Ali

**Affiliations:** 1 Riphah Institute of Pharmaceutical Sciences, Riphah International University, Islamabad, Pakistan; 2 Department of Biomedical Science, Pak-Austria Fachhochule: Institute of Applied Science and Technology, Mang Haripur, KPK Pakistan; 3 Department of Pharmacy, Iqra University, Islamabad, Pakistan; 4 Department of Pharmacy, Kohat University of Science and Technology, Kohat, Pakistan

**Keywords:** L-carnitine, Pruritus, Nitric oxide, Anti-oxidant, Anti-inflammatory

## Abstract

**Background:**

Pruritus, or itching, is a distressing symptom associated with various dermatological and systemic diseases. L-carnitine (βeta hydroxy-γ-tri methyl amino-butyric acid), is a naturally occurring substance, it controls numerous physiological processes. The present research aims to identify L-carnitine for its anti-pruritic effect via nitric oxide-dependent mechanism.

**Methods:**

Chloroquine-induced pruritus serves as an experimental model to investigate possible therapeutic interventions. In this study, we evaluated the efficacy of L-carnitine in combating oxidative stress, nitric oxide, and inflammatory cytokines in a chloroquine-induced pruritus model.

**Results:**

L-carnitine treatment significantly reduced scratching behavior compared to the disease group (^***^P < 0.001 vs. chloroquine group), indicating its antipruritic potential. The markers of oxidative stress, GST, GSH, Catalase, and LPO were dysregulated in the disease model, but administration of L-carnitine restored GST, GSH, and Catalase levels and decreased LPO levels (^***^P < 0.001 vs. chloroquine group), thereby alleviating oxidative stress. L-carnitine also reduced nitric oxide synthase (NOS) activity, suggesting that it modulates nitric oxide signaling pathways involved in pruritus. In addition, L-carnitine lowered levels of pro-inflammatory cytokines such as tumor necrosis factor-alpha (TNF-α), inflammatory marker nuclear factor kappa B (p-NFκB) and also reduces an inflammatory enzyme, cyclooxygenase-2 (COX-2), determined by ELISA (Enzyme-Linked Immunosorbent Assay) (^***^P < 0.001 vs. chloroquine group). It downregulates nNOS mRNA expression confirmed by real-time polymerase chain reaction (RT-PCR).

**Conclusion:**

These findings highlight the therapeutic effects of L-carnitine in alleviating chloroquine-induced pruritus.

## Background

The skin is an organ that acts as a physical barrier between the body and the outside world and is essential for preserving healthy physiologic conditions. Even though the skin is constantly exposed to varying physical, chemical, and microbiological insults, the defense system maintains skin homeostasis by activating a variety of immune cells [1]. These immunological responses have coevolved for the survival of the host, but when they are dysregulated, they lead to sensory dysfunction, especially in the form of a chronic itch, which is an uncomfortable and emotional experience and an itchy feeling that makes you want to scratch [2]. Itchy skin or pruritus is an uncomfortable sensation that makes feel a scratch and desire to rub, to ease it. It can be either because of some underlying dermatological skin disease presentation or some primary diseases of the skin [3]. Itchy skin is observed during the process of inflammation, some metabolic diseases, psychiatric illnesses, drug abuse, stress and various malignancies as well. Pruritus is among the 50 most prevalent diseases worldwide. It’s a common symptom that leads to 7 million ambulatory visits in the US. In Pakistan, the prevalence of pruritus is 70% associated with different diseases [4].

There are various mechanisms involved in the process of itching including significant release of substance P (a neuropeptide), prostaglandin release, and histamine release from mast cells. However, it is also controlled naturally inside our body by various regulatory processes [5].

Nitric oxide has a crucial role in normal physiological function as well as in a variety of pathological disorders. A neurotransmitter called nitric oxide (NO) is considered to be a mediator of pruritus [6]. It contributes to the etiology of pruritus. Mast cells, fibroblasts, keratinocytes, macrophages, primary sensory neurons and endothelial cells are examples of skin cells that may emit NO [7]. Nitrate concentrations in the serum are higher in atopic dermatitis patients [8]. Experimental findings indicates that regular dose of N-nitro L-arginine methyl ester (L-NAME) reduces itch in AD subjects by 80% [9]. The pruritic condition of psoriasis causes an increase in the level of NO in the skin. The fact that chloroquine injection causes scratching behavior, which is mediated by the NO pathway, suggests that NO may alter itch in a variety of clinical pruritic diseases [10]. The itch sensation pathways in the spinal cord, brain and C-fiber all include NOS. Scratching-related reactions are brought on by an increase in NO generation caused by intradermal substance P [11].

L-carnitine (βeta hydroxy-γ-tri methyl amino-butyric acid), a naturally occurring substance, is vital to life, controls numerous physiological processes. L-carnitine is synthesized from lysine and methionine, two amino acids, and is essential for metabolism and β-oxidation. By alleviating oxidative stress, lipid peroxidation and generation of ROS (reactive oxygen species), L-carnitine helps to reduce inflammation [12].

Despite numerous modern advancements, there is still no ideal treatment for pruritus. In the world of medicine, there is a great deal of untapped research potential. Furthermore, current therapies only offer short-term relief from these problems; in contrast, natural sources are thought to provide a safer therapeutic option that offers longer-lasting comfort. Herbal extracts and natural medicinal compounds make up 61% of all pharmaceuticals sold worldwide and are often regarded as essential components of modern medicine [13].

The present study’s goal was to identify L-carnitine for its anti-pruritic effect via nitric oxide dependent mechanism.

## Material and methods

### Chemicals

L-carnitine was purchased from UAE (carnipure trademark of Lonza, Switzerland). 3, 3-diaminobenzidine peroxidase (CAS# 91-95-2), Avidin Biotin Complex (CAS 1405-69-2), 5, 5′-dithiobis (2-nitrobenzoic acid) (DTNB, #D8130, PubChem ID: 24894189), trichloroacetic acid (TCA, #T6399, PubChem ID: 24,900,373), L-arginine (CAS# 74-79-3 SIGMA), L-NAME (CAS# 51298-62-5), Aminoguanidine (CAS# 1937-19-5), and 7-NI (CAS# 2942-42-9) were purchased from Sigma-Aldrich (USA, St. Louis, MO). Purchasing of chloroquine injections was done by the local pharmaceutical industry. Secondary antibodies were obtained from Abcam UK. p-NFκB ELISA kit (Cat NO.PRS-20640Mo), TNF-α ELISA kit (Cat NO. PRS-30651Ra), and COX-2 ELISA kit (Cat NO. PRS-30205Ra) were purchased from Nanjing Pars Biochem CO., Ltd China. Analytical-grade chemicals were used in the study.

### Animals

Adult mice weighing about 25–30 g of either sex, held in Riphah Institute of Pharmaceutical Sciences (RIPS) animal house were used in the experiment. All experiments were performed according to the guidelines for using laboratory animals by ARRIVE guidelines (2019) and the US National Institutes of Health, as approved by the Research and Ethics Committee of Riphah Institute of Pharmaceutical Sciences, Riphah International University (Reference no: REC/RIPS/2022/06). A 12/12 hr light-dark cycle and relative humidity at 55% ± 5% was maintained and room temperature was controlled at 25 °C ± 1 °C. Food and water were provided at libitum. All methods carried out in this study were performed following relevant guidelines and regulations. All animals were anesthetized using a cocktail of xylazine (9 mg/kg) and ketamine (90 mg/kg) intraperitoneally and were euthanized by cervical dislocation following AVMA guidelines. All efforts were made to minimize their suffering.

### Chloroquine-induced scratching

Mice were divided into different groups (n = 5–7/group) and their skin were shaved at the nape of the neck for intradermal injection. Control group was given normal saline (10 mL/kg, i.p.), where as standard group was treated with cetirizine (0.25 mg/kg) and chloroquine (CQ) at the dose of 400 ug intradermally was given after 4 hr. CQ was injected to induce pruritus after the administration of different doses of (LC) L-Carnitine (50, 75, 100 and 200 mg/kg; p.o), L-NAME (1 mg/kg, i.p.), L-arginine (100 mg/kg, i.p.), amino guanidine (200 mg/kg, i.p.) [14]. 7-nitroindazole (7-NI) 1 nmoles/site was injected intradermally at the same site and time when CQ was injected. L-NAME (1 mg/kg), L-arginine (100 mg/kg) and AG (amino guanidine 200 mg/kg) were administered 15 min after L-carnitine [15].

### Behavioural studies

All of the animals were individually housed in an acrylic cage (10 × 10 × 13 cm) at 25 °C for at least an hour before the tests to allow for acclimatisation. After CQ injection, their behaviour was recorded using a video camera. The recorded video was used later to count the scratchings by the mice using the hind paws at the site where CQ was injected. The average duration of a mouse’s scratching is one second and multiple motions were counted as one scratching session. The cleaning action of mice by forelimb was considered as normal behaviour. Video was recorded for 1 hr. The mice scratch the shaved area once was considered as 1 bout or single action/scratch. With a gap of more than 1s between each scratch, number of scratches were counted [15].

### Sample collection and preparation for molecular studies

After behavoural studies the animals were anesthetized under anesthesia and euthanized. Following euthanization, skin tissues were collected from the shaved area at the nape of the neck onto an ice-cold glass plate at 24 h after the behavioural analysis. The skin tissues were isolated and divided into two groups: one group was retained in 10% formalin solution for immunohistochemistry and histopathological investigation and the other group was preserved at −80 °C for biochemical processing. Using 0.1 M sodium phosphate buffer (pH 7.4) with phenylmethylsulfonyl fluoride (PMSF) as a protease inhibitor, skin tissue samples were homogenized at 4000 rpm x g for 10 min at 4 °C and then centrifuged for atleast 30 mins and then the resultant supernatant was collected. This supernatant was then employed for numerous biochemical experiments i.e anti-oxidant profile, ELISA and RT-PCR [16].

### Anti-oxidant profile

After completion of doses, all animals were sacrificed and their shaved skin tissues were removed. At 4000 rpm the skin samples were first homogenized and then centrifuged for at least 30 minutes. The resulting supernatant was tested for the presence of glutathione (GSH), glutathione S-transferases (GST), catalase and lipid peroxidase (LPO) to determine its antioxidant effect. A yellow end product known as 2-nitro-5- thiobenzoic acid was generated by oxidising GSH and DTNP and its absorbance at 412 nm was measured. Values for GSH were represented in micromoles per milligram of proteins [13]. By the CDNB conjugate formation, GST level was evaluated and at 340 nm the absorbance was measured. The activity of GST was calculated and represented in µmoles of CDNB conjugate/min/mg of proteins [17]. The Degradation of H_2_O_2_ measured catalase activity. Absorbance was measured at 240 nm. The values of Catalase were represented in µmoles H_2_O_2_/min/mg of proteins [18]. Malondialdehyde (MDA), a sign of cell damage or a marker of lipid peroxidation that is produced as a by-product, was identified using the thiobarbituric acid reactive substance (TBARS) assay. Absorbance was measured at 532 nm. LPO was measured as nmoles/min/mg protein TBARS [19].

### Hematoxylin and eosin (H&E) staining

Mice skin tissues were utilized for the morphological analysis. 10% paraformaldehyde was used to fix the skin tissues. Tissues were sliced using a rotary microtome and stained with hematoxylin and eosin (H&E). In each group, 5 microscopic images were captured and examined for epidermis skin layer damage, thickness, deformed cells and vacuolization [20].

### Immunohistochemistry (IHC) investigation

Three distinct absolute xylenes were used to deparaffinize tissue sections mounted on slides and they were rehydrated with ethyl alcohol in a range of concentrations (from 100% [absolute] to 70%). The slides were then kept in 0.01 M phosphate-buffered saline (PBS) for 10 min after being rinsed with distilled water. The slides were treated with appropriate biotinylated secondary antibodies for 2 h after the antigen retrieval stage, then for 1 h with Avidin-biotin complex (ABC) reagents (Standard Vectastain ABC Elite Kit; Vector Laboratories, Burlingame, CA, United States) at the ideal room temperature. The sections were cleaned in PBS, stained with 3,3′-Diaminobenzidine (DAB) solution, dehydrated in graded ethanol solutions (80, 90 and 100%), fixed in xylene, cover-slipped with a mounting medium and allowed to dry by air. The outcomes were examined with a high-end digital photomicroscopy equipment attached to an Olympus light microscope (Japan). Immunohistochemical TIF pictures were captured using a light microscope (5 images per plate). Using ImageJ software, the expression levels of phosphorylated nuclear factor-kappa (p-NFκB), tumor necrosis factor (TNF-α), COX-2, iNOS, and nNOS antibodies were determined [21, 22].

### Enzyme-linked immunosorbent assay (ELISA)

ELISA of p-NFκB, TNF-α and COX-2 were conducted according to the manufacturers instructions. A suitable quantity of skin tissue (50 mg) was first homogenized and the supernatant after centrifugation (at 4000 rpm for 30 min) was collected. BCA method was used to obtain total protein concentration in each group. The supernatant was analysed for TNF-α, p-NFκB and COX-2 using an ELISA kits [23].

### Real time polymerase chain reaction (RT-PCR)

Following homogenization of the skin tissues (n = 3), total ribonucleic acid (RNA) was extracted using the trizol technique in accordance with the manufacturer’s instructions. Reverse transcriptase was used to generate cDNA from 1 to 2 µg of total RNA, and real-time PCR was used to amplify the cDNA using a thermocycler. The levels of GAPDH were used to normalize the mRNA expression. 2^^ΔΔ-CT^ method for real-time quantitative PCR was used to calculate the relative gene expression [24]. Primer sequences for nNOS and GAPDH are as follows:

GGTGGAGATTAACATTGCTGTCCTA (nNOS forward)

TTCTCCATGTGTTTGATGAAGGA (nNOS reverse)

CAACTCCCTCAAGATTGTCAGCAA (GAPDH forward)

GGCATGGACTGTGGTCATGA (GAPDH reverse)

### Statistical analysis

The data was presented as mean ± SEM. By using GraphPad Prism 8 statistical parameters were applied, one-way analysis of variance (ANOVA) with post-hoc Tukey’s test. *P* < 0.05 was considered statistically significant.

## Results

### Effect of L-carnitine (LC) and cetrizine on chloroquine induced scratching

**Fig. 1:**
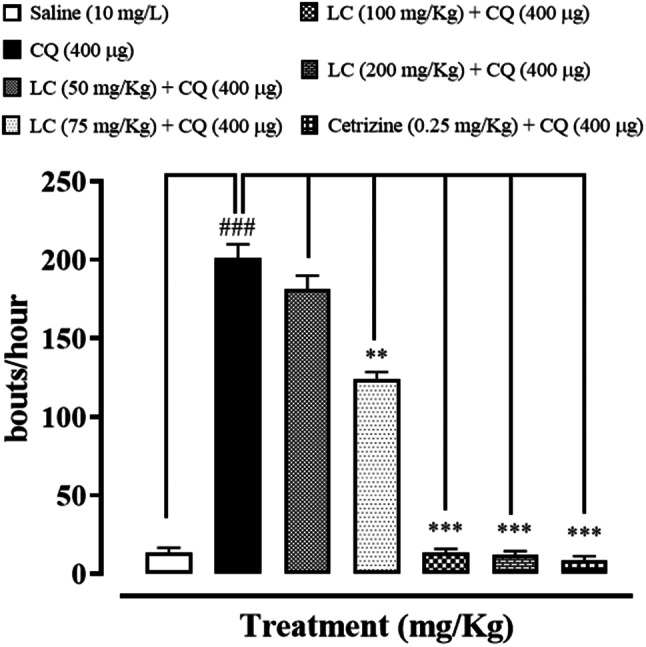
Effects of different doses of (LC) L-Carnitine (50, 75, 100 and 200 mg/kg p.o) against chloroquine-induced scratching in mice skin tissue. Values expressed as mean ± SEM (n = 5). One-way ANOVA with post hoc Tukey’s test. ^###^*P* < 0.001 vs. saline group, ^**^P < 0.01, ^***^P < 0.001 vs. chloroquine group (CQ)

In first experiment different doses of LC (50, 75, 100, 200 mg/kg) were given orally. A significant decrease in scratching activity of mice were observed at doses of 100 and 200 mg/kg (^***^P < 0.001), induced by chloroquine (400 µg, intradermally) as shown in Fig. 1.

### Anti-scratching effect of (LC) L-carnitine, NOS precursor L-arginine and NOS inhibitors

**Fig. 2:**
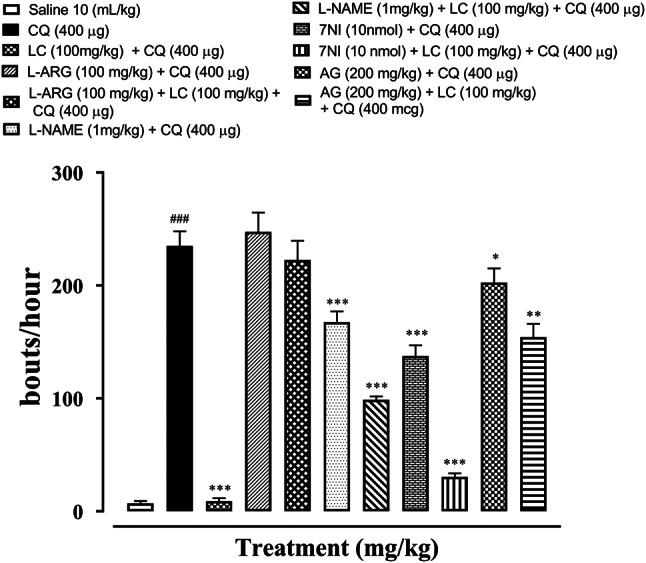
Effect of L-carnitine (LC), L-arginine (L-ARG), N-nitro-L-arginine methyl ester (L-NAME), 7-nitroindazole (7-NI) and aminoguanidine (AG) against chloroquine-induced scratching in mice skin tissue. Values expressed as mean ± SEM (n = 5). One-way ANOVA with post hoc Tukey’s test. ^###^*P* < 0.001 vs. saline,^*^P < 0.05, ^***^P < 0.001 vs. chloroquine group (CQ)

To determine the effect of nitric oxide pathway on the anti-pruritic activity, L-arginine (NO precursor, 100 mg/kg) were administered, it antagonizes the anti-scratching effect of LC. L-arginine when given with CQ 400 µg potentiated its scratching effect and boosted the number of scratches as compared to the disease group. L-NAME (a nonspecific inducible NOS inhibitor) along with LC decreases the pruritic effect of chloroquine (^***^P < 0.001 vs. CQ group). To evaluate the role of nNOS in CQ induced pruritus, 7-nitroindazole (7-NI) was also administered intradermally at a dose of 1 nmol/site. 7-NI significantly enhanced the antipruritic effect of L-carnitine (^***^P < 0.001 vs. CQ group). To evaluate the role of iNOS, its inhibitor, Aminoguanidine (AG) was administered, it increases the LC anti-scratching effect to some extent (^**^P < 0.01 vs. CQ group) (Fig. 2).

### Effect on oxidative stress markers

**Fig. 3:**
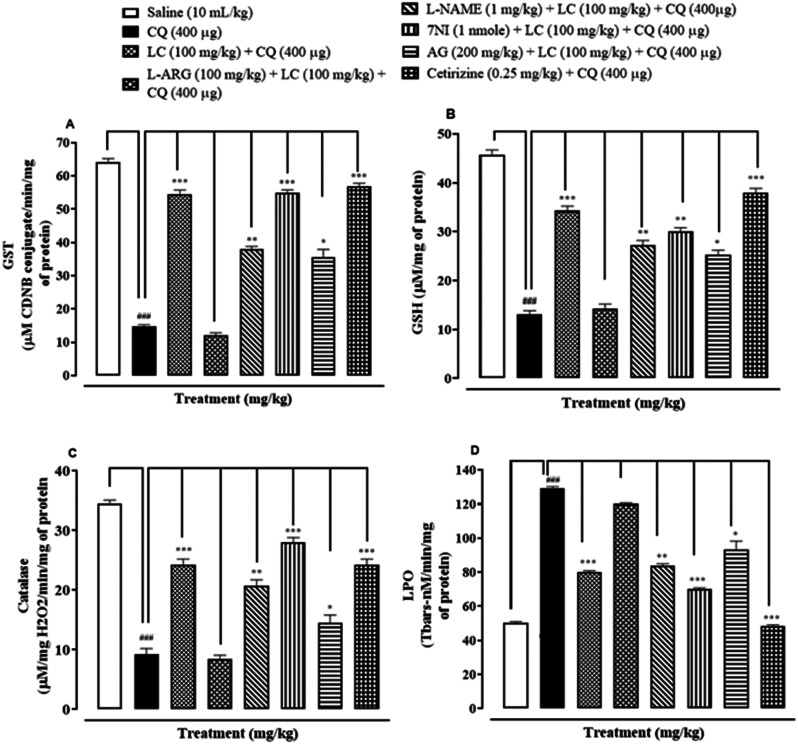
Effect of L-carnitine (LC), L-arginine (L-ARG), N-nitro-L-arginine methyl ester (L-NAME), 7-nitroindazole (7-NI), aminoguanidine (AG) and cetirizine against glutathione sulfotransferases (GST), reduced glutathione (GSH), catalase and lipid peroxidase (LPO) in chloroquine-induced scratching of mice skin tissues. Values expressed as mean ± SEM (n = 5). One-way ANOVA with post hoc Tukey’s test. ^###^*P* < 0.001 vs. saline group, ^*^P < 0.05,^**^P < 0.01, ^***^P < 0.001 vs. chloroquine group (CQ)

To assess whether L-carnitine (LC) confers protection against chloroquine induced oxidative stress, we measured various levels of antioxidants in skin tissues. The levels of GST, GSH, CAT and LPO were measured. CQ and NOS precursor L-Arginine treated groups demonstrated significant reduced levels of GST, GSH and CAT (^###^*P* < 0.001 vs saline, Fig. 3A–C), although LPO was increased (^###^*P* < 0.001 vs saline, Fig. 3D). In contrast, LC (100 mg/kg) was able to restore the levels of these antioxidants (GST ^***^P < 0.001; GSH ^***^P < 0.001; CAT ^***^P < 0.001 vs CQ), while LPO was reduced (^***^P < 0.001 vs. CQ) as shown in Fig. 3. Pre-treatment of NOS inhibitors L-NAME, AG, 7-Ni and cetirizine further restored the levels of GST, GSH, Catalase and reduced LPO (^*^P < 0.05,^**^P < 0.01, ^***^P < 0.001 vs. CQ) (Fig. 3).

### Histopathological examination

**Fig. 4:**
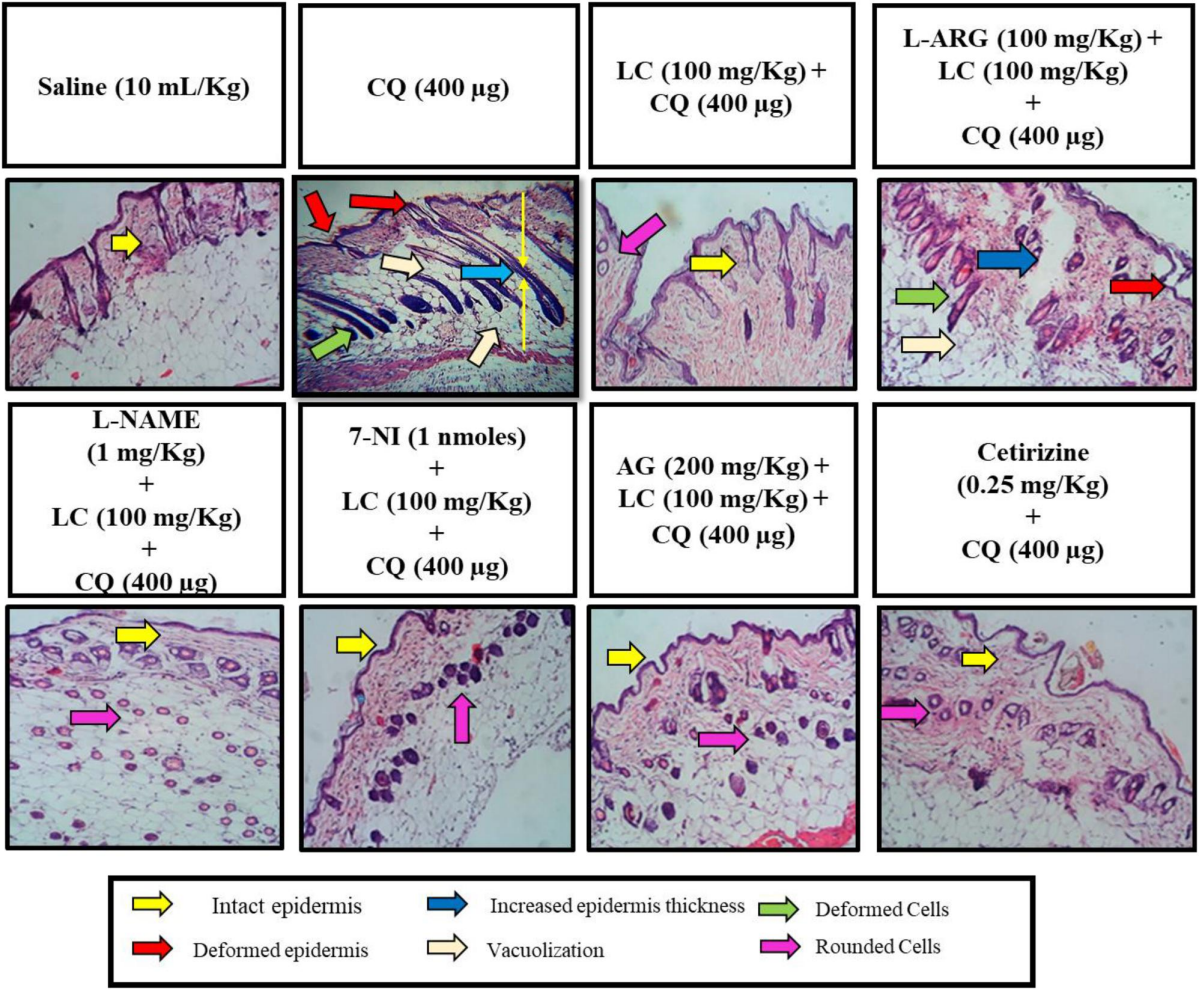
Effect of L-carnitine (LC), L-arginine (L-ARG), N-nitro-L-arginine methyl ester (L-NAME), 7-nitroindazole (7-NI) and aminoguanidine (AG) against chloroquine-induced scratching in mice skin tissue, using hematoxylin and eosin staining (H & E) histopathological technique. Bar 50 µm, magnification 10x. Saline group showing normal histological features. Chloroquine and L-arginine group shows marked histopathological deformities by loss of skin architecture, integrity and necrosis. LC, L-NAME, 7-NI, AG and cetirizine shows marked improvement in terms of epidermis deformity from moderate to mild changes

In saline group normal skin tissues and architecture were visible without any pathological alterations. Chloroquine (CQ) and L-Arginine (100 mg/kg) treated tissues exhibited severe damage with disruption, vacuolization, thickness of epidermis (first skin layer) and deformed cells. Skin tissues from mice treated with LC (100 mg/kg), NOS inhibitors (L-NAME, 7NI and AG), and cetirizine (0.25 mg/kg) exhibits tissue regeneration and restoration with much improved cellular architecture (Fig. 4).

### IHC analysis

**Fig. 5:**
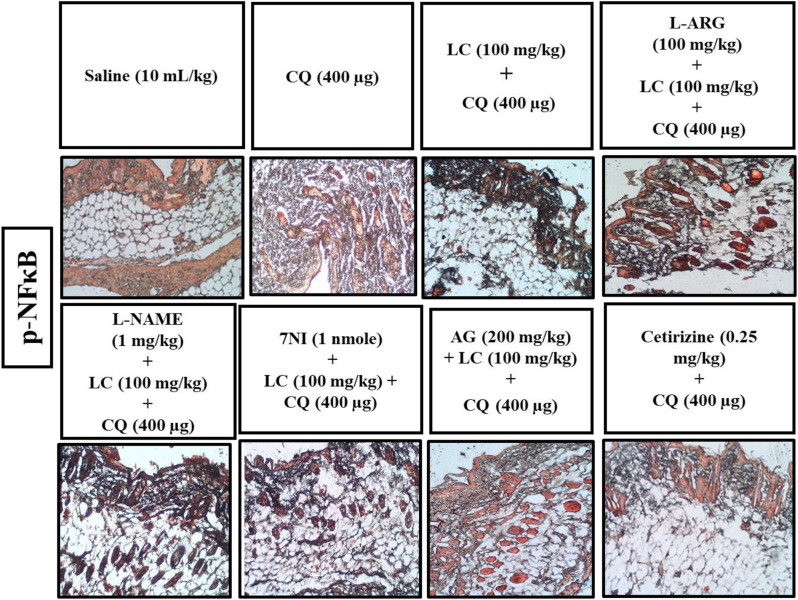
Effect of L-carnitine (LC), L-arginine (L-ARG), N-nitro-L-arginine methyl ester (L-NAME), 7-nitroindazole (7-NI), aminoguanidine (AG) and cetirizine against apoptotic marker nuclear factor kappa B (p-NFκB) in mice skin tissue, using immunohistochemical technique. Bar scale 50 µm, magnification 10x

**Fig. 6:**
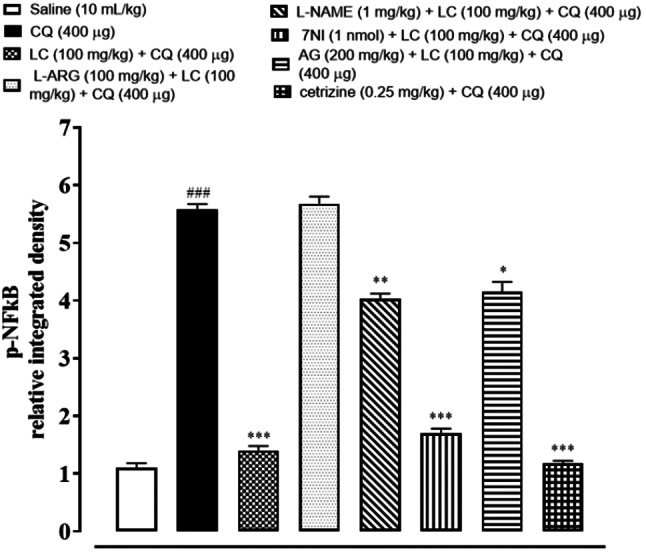
Inhibitory effect of L-carnitine (LC), N-nitro-L-arginine methyl ester (L-NAME), 7-nitroindazole (7-NI), aminoguanidine (AG) and cetirizine against nuclear factor kappa B (p-NFκB) expression in mice skin tissue, using immunohistochemical technique. Values expressed as mean ± SEM (n = 5). One-way ANOVA with post hoc Tukey’s test. ^###^*P* < 0.001 vs. saline group, ^*^P < 0.05, ^**^P < 0.01, ^***^P < 0.001 vs. chloroquine group

**Fig. 7:**
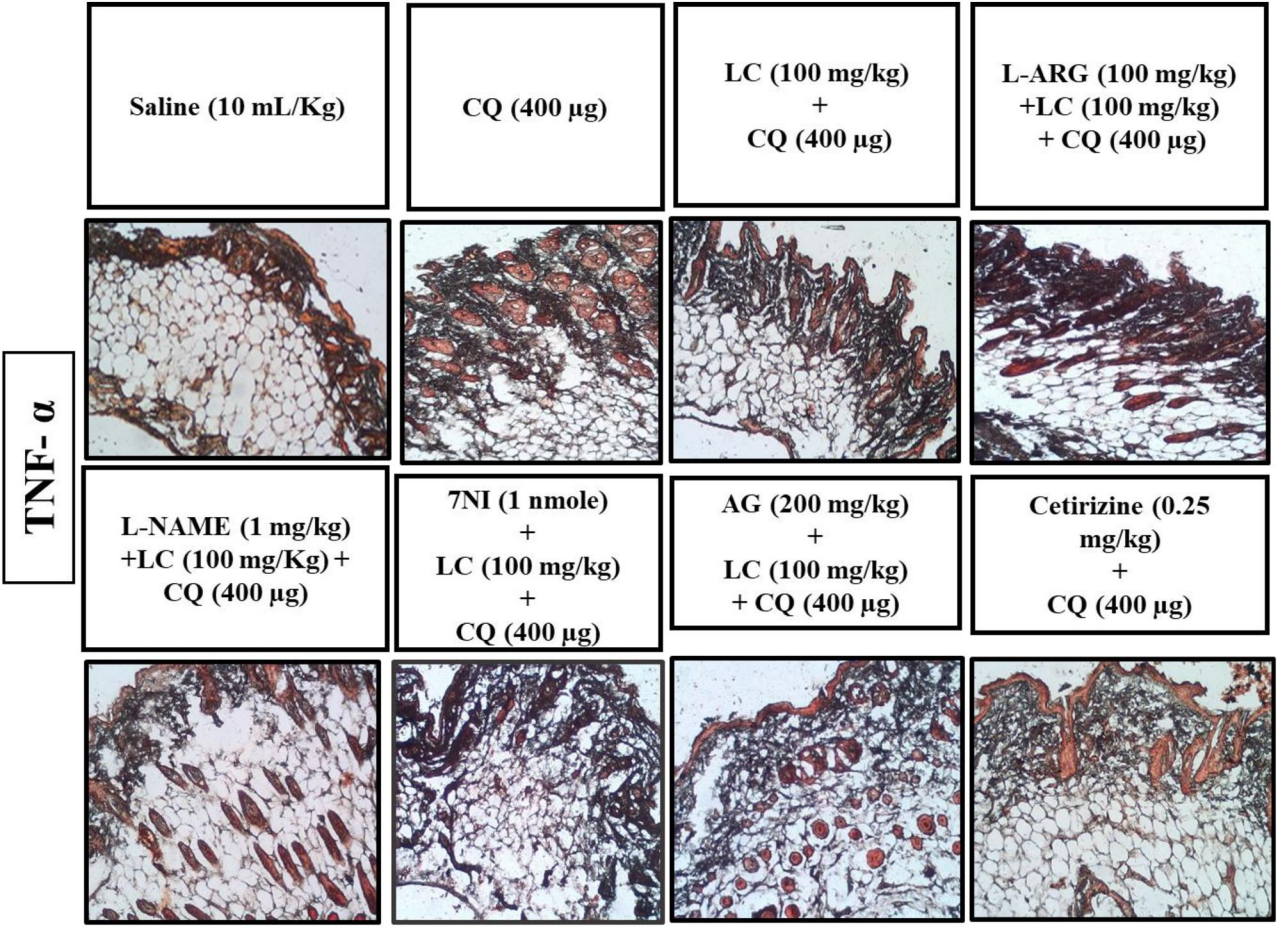
Effect of L-carnitine (LC), L-arginine (L-ARG) N-nitro-L-arginine methyl ester (L-NAME), 7-nitroindazole (7-NI), aminoguanidine (AG) and cetirizine against apoptotic marker tumor necrosis factor (TNF-α) of mice skin tissues, using immunohistochemical technique. Bar size 50 µm, magnification 10x

**Fig. 8:**
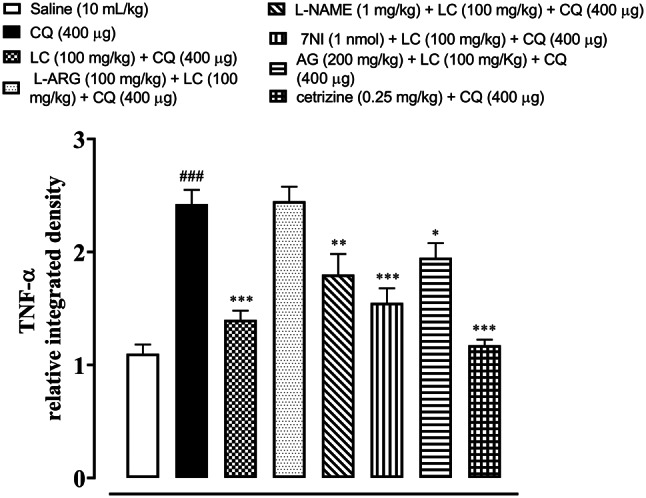
Inhibitory effect of L-carnitine (LC), N-nitro-L-arginine methyl ester (L-NAME), 7-nitroindazole (7-NI), aminoguanidine (AG) and cetirizine against tumor necrosis factor alpha (TNF-α) expression in mice skin tissues, using immunohistochemical technique. Values expressed as mean ± SEM (n = 5). One-way ANOVA with post hoc Tukey’s test. ^###^*P* < 0.001 vs. saline group, ^*^P < 0.05, ^**^P < 0.01, ^***^P < 0.001 vs. chloroquine group

**Fig. 9:**
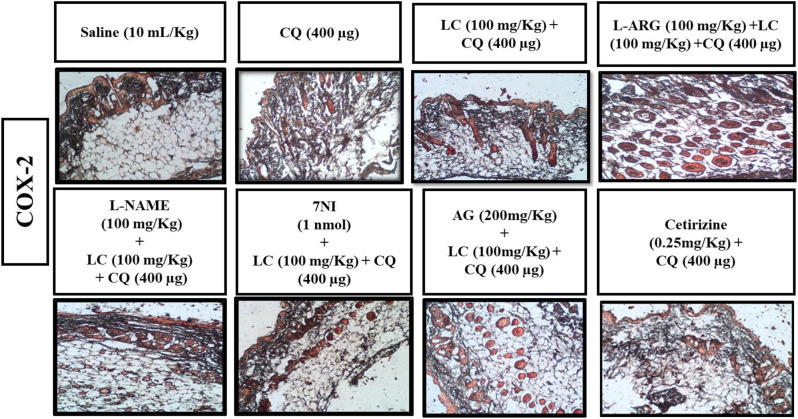
Effect of L-carnitine (LC), L-arginine (L-ARG) N-nitro-L-arginine methyl ester (L-NAME), 7-nitroindazole (7-NI), aminoguanidine (AG) and cetirizine against apoptotic marker cyclooxygenase-2 (COX-2) of mice skin tissues, using immunohistochemical technique. Bar size 50 µm, magnification 10x

**Fig. 10:**
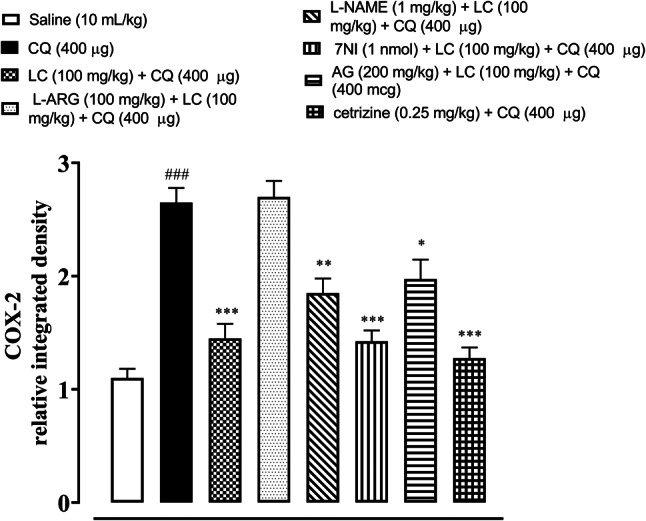
Inhibitory effect of L-carnitine (LC), N-nitro-L-arginine methyl ester (L-NAME), 7-nitroindazole (7-NI), aminoguanidine (AG) and cetirizine against cyclooxygenase-2 (COX-2) expression of mice skin tissues, using immunohistochemical technique. Values expressed as mean ± SEM (n = 5). One-way ANOVA with post hoc Tukey’s test. ^###^*P* < 0.001 vs. saline group, ^*^P < 0.05, ^**^P < 0.01, ^***^P < 0.001 vs. chloroquine group

**Fig. 11:**
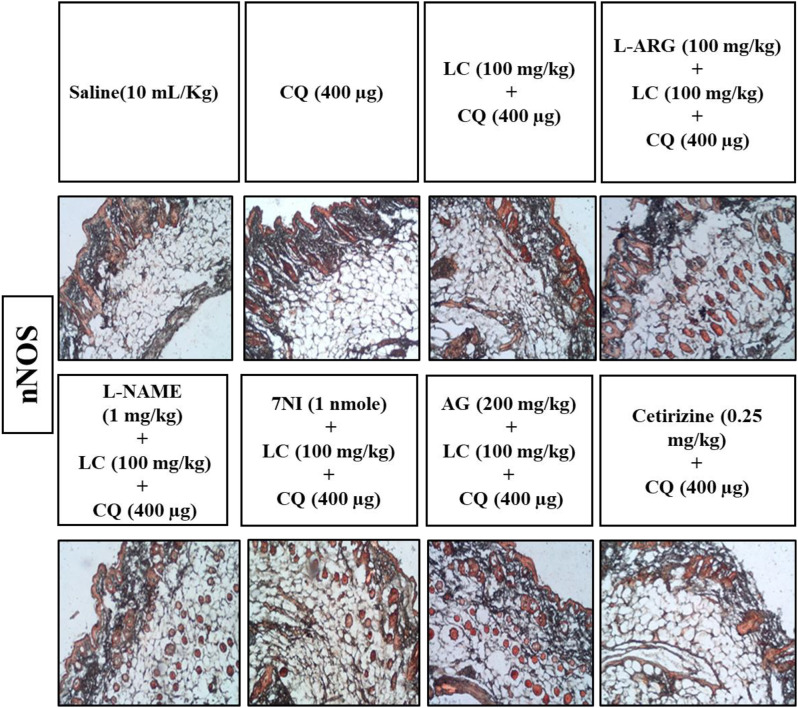
Effect of L-carnitine (LC), L-arginine (L-ARG), L-NAME (N-nitro-L-arginine methyl ester), 7-nitroindazole (7-NI), aminoguanidine (AG) and cetirizine against apoptotic marker n-NOS in mice skin tissues, using immunohistochemical technique. Bar scale 50 µm, magnification 10x

**Fig. 12:**
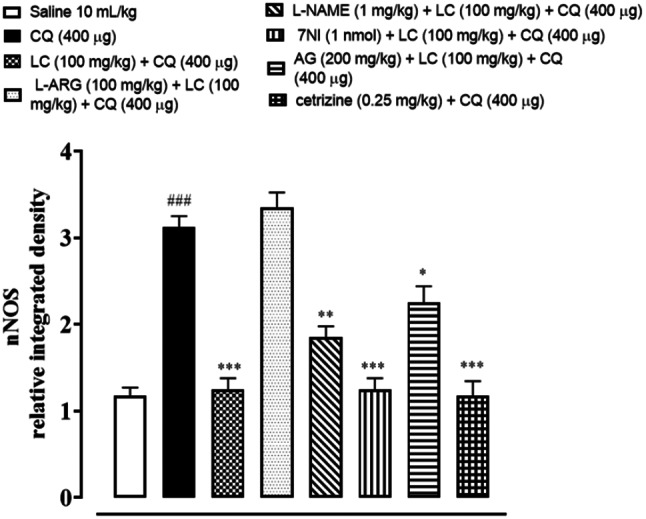
Inhibitory effect of L-carnitine (LC), L-NAME (N-nitro-L-arginine methyl ester), 7-nitroindazole (7-NI), aminoguanidine (AG) and cetirizine against nNOS expression in mice skin tissues, using immunohistochemical technique. Values expressed as mean ± SEM (n = 5). One way ANOVA with post hoc Tukey’s test ^###^*P* < 0.001 vs. saline group,^*^P < 0.05, ^**^P < 0.01, ^***^P < 0.001 vs. chloroquine group

**Fig. 13:**
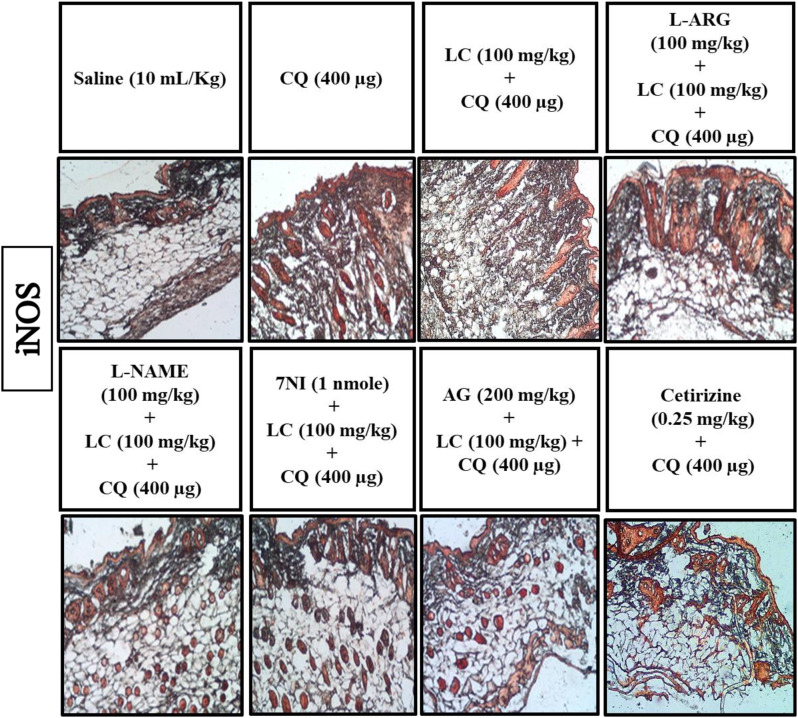
Effect of L-carnitine (LC), L-arginine (L-ARG), N-nitro-L-arginine methyl ester (L-NAME), 7-nitroindazole (7-NI), aminoguanidine (AG) and cetirizine against apoptotic marker iNOS in mice skin tissues, using immunohistochemical technique. Bar scale 50 µm, magnification 10x

**Fig. 14:**
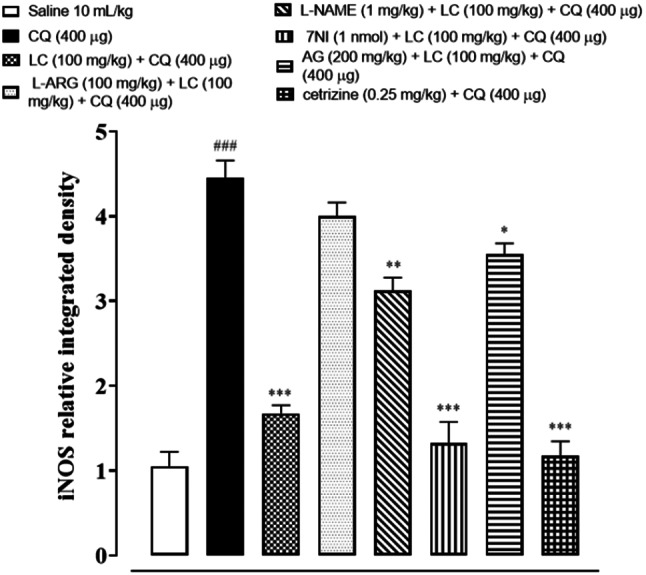
Inhibitory effect of L-carnitine (LC), N-nitro-L-arginine methyl ester (L-NAME), 7-nitroindazole (7-NI), aminoguanidine (AG) and cetirizine against iNOS expression in mice skin tissues, using immunohistochemical technique. Values expressed as mean ± SEM (n = 5). One way ANOVA with post hoc Tukey’s test ^###^*P* < 0.001 vs. saline group, ^*^P < 0.05, ^**^P < 0.01, ^***^P < 0.001 vs. chloroquine group

The skin tissues were subjected to immunohistochemical analysis to estimate the degree of expression of pro-inflammatory and inflammatory markers as p-NFκB, TNF-α, COX-2, also for neuronal nitric oxide synthase (nNOS) and inducible nitric oxide synthase (iNOS). As shown in Figs. 5, 6, 7, 8, 9, 10, 11, 12, 13 and 14, in mice skin tissues the levels of p-NFκB, TNF-α, COX-2, nNOS and iNOS were increased in CQ (400 µg) and L-ARG treated groups (^###^*P* < 0.001 vs. saline group). Treatment with LC (100 mg/kg) and Cetirizine (^***^P < 0.001 vs. CQ) decreased the expression of p-NFκB, TNF-α, COX-2, nNOS and iNOS (Figs. 5, 6, 7, 8, 9, 10, 11, 12, 13 and 14). A significant amelioration in p-NFκB, TNF-α, COX-2, nNOS and iNOS expression was seen after co-treatment of LC with L-NAME (^**^P < 0.01 vs. CQ group), AG (^*^P < 0.05 vs. CQ group) and 7Ni (^***^P < 0.001 vs. CQ group) as shown in Figs. 5, 6, 7, 8, 9, 10, 11, 12, 13 and 14 respectively.

### Effects on inflammatory markers

**Fig. 15:**
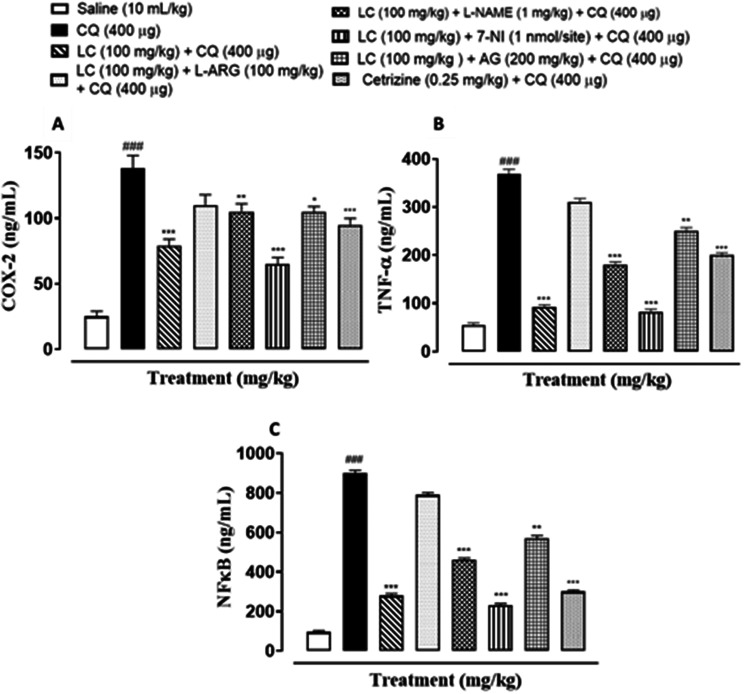
Inhibitory effect of L-Carnitine (LC), N-nitro-L-arginine methyl ester (L-NAME), 7-nitroindazole (7-NI), amino guanidine (AG) and cetirizine against (**A**) cyclooxygenase-2 (COX-2), (**B**) tumor necrosis factor (TNF-α) and (**C**) nuclear factor kappa B (p-NFκB) levels in chloroquine-induced scratching in mice skin tissues, using enzyme-linked immunosorbent assay technique. Values expressed as mean ± SEM (n = 5). One-way ANOVA with post-hoc Tukey’s test. ^###^*P* < 0.001 vs. saline group. ^*^P < 0.05, ^**^P < 0.01, ^***^P < 0.001 vs. chloroquine group

The skin tissues supernatant were subjected to ELISA to estimate the degree of expression of pro-inflammatory and inflammatory markers as p-NFκB, TNF-α and COX-2. In skin tissues the levels of p-NFκB, TNF-α and COX-2 were increased in CQ and L-ARG treated groups (^###^*P* < 0.001 saline group). Treatment with LC, L-NAME, 7NI, AG and cetirizine decreased the levels of these inflammatory markers (^*^P < 0.05, ^**^P < 0.01, ^***^P < 0.001 vs. chloroquine group) as shown in Fig. 15.

### Quantification of mRNA level

**Fig. 16:**
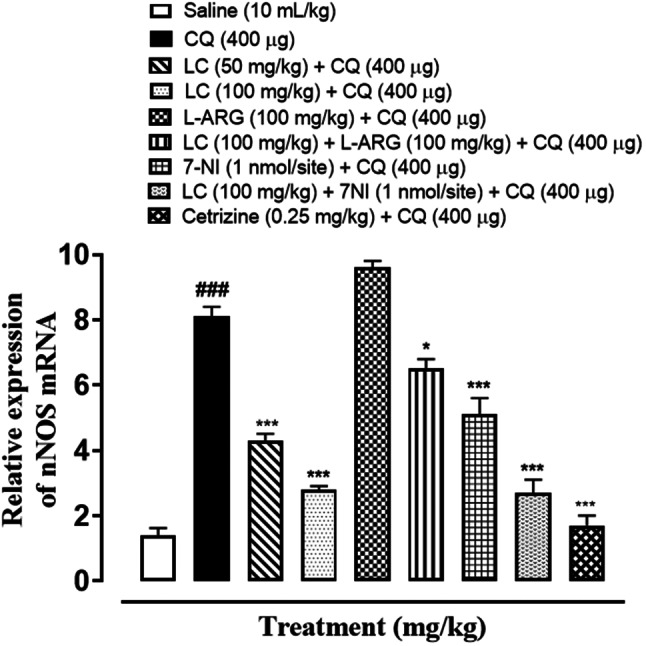
Inhibitory effect of L-carnitine (LC), 7-nitroindazole (7-NI) and cetirizine against nNOS mRNA expression in chloroquine-induced scratching of mice skin tissue, using real-time polymerase chain reaction (RT-PCR) technique. One-way ANOVA followed by post-hoc Tukey’s test. Values expressed as mean ± SEM (n = 3). ^###^*P* < 0.001 vs. saline group, ^*^P < 0.05, ^**^P < 0.01, ^***^P < 0.001 vs. chloroquine group

RT-PCR determined the fold expression of Neuronal Nitric Oxide Synthase (nNOS) in CQ-induced scratching. In CQ and L-ARG treated groups the expression of nNOS mRNA levels were increased (^###^*P* < 0.001 vs. saline group). Treatment with LC (100 mg/kg), 7-NI and cetirizine (0.25 mg/kg) decrease n-NOS mRNA levels (^*^P < 0.05, ^**^P < 0.01, ^***^P < 0.001 vs. chloroquine group), as shown in Fig. 16.

## Discussion

A number of pharmaceuticals have been invented to treat various disease symptoms, but they do not address the underlying causes of such diseases and are toxic to be utilized as preventative measures [25]. The importance of naturally occurring compounds in the field of health and medicine is extensive all over evolution. These are used as a single source to treat various injuries and illnesses [26]. L-Carnitine (βeta hydroxy-γ-tri methyl amino-butyric acid) is a naturally occurring compound that is essential for life as it regulates various physiological function. L-Carnitine produce from the amino acids (lysine and methionine) synthesis and plays a vital role in metabolism and β-oxidation [12]. L-carnitine is involved in reducing inflammation by targeting oxidative stress, ROS (reactive oxygen species) generation and by decrease in lipid peroxidation [27].

Nitric oxide synthases (NOS) expressed with three isoforms, including iNOS (inducible), eNOS (endothelial) and nNOS (neuronal) are responsible for producing nitric oxide from L-arginine [28]. NO is an important chemical for cellular signaling. It participates in angiogenesis and the development of the nervous system and helps regulate vascular tone, insulin secretion, airway tone, and peristalsis. nNOS, a soluble enzyme, is constitutively expressed in the brain and peripheral nerves [29].

Patients with pruritic conditions including atopic eczema and psoriasis may produce more NO in their skin. CQ can stimulate NOS activity in the skin to produce itch sensation. In the previous studies it was reported that scratching behavior resulted from increase epidermal NO level by nNOS after administration of substance P [30]. The results of current study shows that CQ-induces dose dependent increase in scratching behavior. Non-selective NOS inhibitor (L-NAME) suppress this behavior, indicating the involvement of NO in CQ-induced scratching. Aminoguanidine shows minimal effect on scratching [31]. Regarding the inhibitory effects of 7-nitroindazole as a selective nNOS inhibitor on scratching behavior, it seems that nNOS plays a significant role in this phenomenon. This study reveals that CQ can stimulate NOS activity in the skin to produce itch sensation. Previous studies demonstrated metformin’s antipruritic action via the NO pathway. Subeffective dosages of metformin combined with L-NAME and 7-NI reduced CQ-induced scratching behavior, whereas L-arginine reversed the effect, indicating the involvement of the NO pathway in pruritus [14, 15]. CQ involves NO pathway for the itch manifestation, a histamine independent itch response [32]. Different NOS inhibitors L-NAME, 7NI and AG were given systemically. NOS prvecursor L-ARG was also given. Effective dosages of L-carnitine or 7-NI reduced the increased cutaneous NO caused by CQ. Therefore, it appears that L-carnitine’s activity is involved in inhibition of the intradermal NO pathway, probably through nNOS, whereas L-ARG reverses it.

Previous studies indicate L-carnitine possess free radical scavenging activity [33], which may be responsible for its effectiveness as anti-pruritic agent. Antioxidant activity is further validated this by LPO, catalase, GST and GSH assay. CQ and CQ with L-ARG vigorously increases the LPO levels while decreases GST, GSH and catalase levels. GSH, GST and catalase levels increased whereas LPO levels declined when LC was administered alone or in conjunction with NOS inhibitors (L-NAME, 7NI, and AG). These findings suggested that antioxidants alleviate itching through reducing oxidative stress [34].

Histopathological studies reveal that CQ group and CQ combined with L-ARG group showed vigorous morphological changes i.e., deformed epidermis and cells, increased epidermal thickness, vacuolization and loss of membrane integrity. In our treatment groups (L-carnitine alone and combined treatment with L-NAME, 7NI and AG), these morphological changings and cell necrosis were restored whereas the significant effect were seen in 7NI treatment group.

COX-2, TNF-α and p-NFκB are considered to be a main target in inflammatory skin disorders and are mostly utilized in pharmacological therapy [18]. Their expression is mostly increased in inflammatory processes. The itching process is stimulated by NO overproduction caused by enhanced n-NOS and iNOS expression [6]. ELISA and IHC were carried out to analyze the above mentioned parameters. In contrast to the control group, IHC analysis showed that p-NFκB, TNF-α, COX-2, n-NOS and iNOS were considerably overexpressed in skin tissues. When treated with LC, NOS inhibitors and cetirizine these expressions were significantly reduced. There was remarkably decreased expression of p-NFκB, TNF-α and COX-2 in treatment group LC combined with 7-NI estimated through ELISA technique. The decrease expression of inflammatory mediators may be linked to its anti-inflammatory property.

RT-PCR study revealed the increase expression of n-NOS mRNA levels in CQ group and L-ARG group and there was no change in control group. While in treatment groups L-carnitine, 7NI and cetirizine significantly reduced expression as compared to disease group indicating the involvement of n-NOS pathway in anti-scratching effect of L-carnitine.

## Conclusions

The present study reveals that L-carnitine possesses anti-oxidant, anti-inflammatory and anti-pruritic effects possibly mediated through nitric oxide pathway.

## Data Availability

Data will be available on request from corresponding author.
